# Emergency general surgery in Italy during the COVID-19 outbreak: first survey from the real life

**DOI:** 10.1186/s13017-020-00314-3

**Published:** 2020-05-24

**Authors:** Alberto Patriti, Gian Luca Baiocchi, Fausto Catena, Pierluigi Marini, Marco Catarci, FACS on behalf of the Associazione Chirurghi Ospedalieri Italiani (ACOI), Di Venere Beatrice, Sallustio Pierluca Nicola Massimo, Siquini Walter, Sorrentino Mario, Polastri Roberto, Armellino Mariano Fortunato, Scatizzi Marco, Giuliani Antonio, Maggioni Dario, Rizzi Andrea, Bonilauri Stefano, Cimino Giuseppe, Contine Alessandro, Borghi Felice, Parisi Amilcare, Longo Graziano, De Luca Stefano, Testa Silvio, Elio Amedeo, Bellochi Raffaele, Boncompagni Michela, Delogu Leonardo Andrea, Gattolin Andrea, Santi Stefano, Agresta Ferdinando, Bottino Vincenzo, Ubiali Paolo, Scuderi Vincenzo, Verzelli Augusto, Cicetti Moreno, Clementi Marco, Annesi Matteo, Stracqualursi Antonio, Sarro Giuliano, Coletta Pietro, Spaziani Alessandro, Pernazza Graziano, Castaldo Pasquale, Spampinato Marcello Giuseppe, Bartoli Alberto, Tirrò Antonino, Cocozza Eugenio, Ferrero Alessandro, Berti Stefano, Perrotta Michele, De Manzini Nicolò, Castagnoli Giampaolo, Celi Daniele, Taglietti Lucio, Vettoretto Nereo, Rabuini Claudio, Santarelli Mauro, Kiss Alberto, Vicentini Roberto, Risio Domenico, Bazzi Piero, Cillara Nicola, Cuticone Giuseppe, Miconi Guglielmo, Cesari Maurizio, Ciaccio Giovanni, Iarrobino Gianfausto, Gambardella Denise, Ceccarelli Graziano, Bordoni Pierpaolo, Isolani Simone Mario

**Affiliations:** 1 Department of Surgery, Azienda Ospedaliera Marche Nord, Ospedale San Salvatore, Piazzale Cinelli 1, Pesaro-Fano, Italy; 2 Department of Clinical and Experimental Sciences, University of Brescia, Brescia, Italy; 3 Emergency Surgery Unit, AOU Parma, Parma, Italy; 4 General Surgery Unit, Azienda Ospedaliera San Camillo-Forlanini, Roma, Italy; 5 General Surgery Unit, Ospedale “C. e G. Mazzoni”, ASUR Marche AV5, Ascoli Piceno, Italy

**Keywords:** Coronavirus, COVID-19, Epidemic, Pandemic, Emergency surgery, Laparoscopy, Management, Resources, Surgery

## Abstract

**Background:**

COVID-19 pandemic has rapidly spread in Italy in late February 2020. Almost all surgical services have been reorganized, with the aim of maintaining an adequate therapeutic path, especially for surgical emergencies. The knowledge of how surgeons dealing with emergency surgery have reacted to the epidemic in the real life can be useful while drafting clinical recommendations.

**Methods:**

Surgeons from multiple Italian regions were invited answering to an online survey in order to make a snapshot of their current behaviors towards COVID-19-positive patients bearing urgent surgical diseases. Questions about institutional rules and personal approach for patient treatment and to limit epidemic spread were included in a 37-item questionnaire.

**Results:**

Seventy-one questionnaires from institutions dealing with emergency surgery were accepted. Participating surgeons were equally subdivided from a geographical point of view, with a large proportion of public (97.2%) and non-academical (91.5%) centers. In 80.3% of cases, the hospitals treated COVID-19 patients; in 69.1% of centers, a change in work plan was necessary, and 33.8% of teams had almost a surgeon infected or in preventive quarantine. The vast majority of surgeons operated only on urgent cases (73.9%), but the number of interventions significantly dropped. Up to 40% of non-traumatic abdominal emergency cases had an unusual delayed treatment. The laparoscopic approach was used in 69.6% of interventions on COVID-19 patients. Strategies to protect health care workers against COVID-19 infection and to identify asymptomatic infected surgeons were suboptimal with respect to the WHO recommendations in 70.4% and 90.2% of centers, respectively. Advanced personal protective equipment for operating room workers was adopted for all surgeries in only 12.7% of centers.

**Discussion:**

This survey confirms that the COVID-19 outbreak is dramatically changing the practice of emergency surgery centers in Italy. Despite the reduction in number, urgent cases were on average more challenging owing to diagnostic delay. Recommendations from the International Scientific Societies are frequently not complied concerning the use of laparoscopic approach, the availability of personal protective equipment in the operating rooms, and the testing of both asymptomatic physicians and patients scheduled for surgery. A further evaluation of the short-term results of these attitudes is warranted to modulate international recommendations.

## Introduction

The COVID-19 epidemic was declared by the World Health Organization (WHO) as a public health emergency on January 30, 2020 [1]. After an initial diffusion in China [2], Italy represents one of the most affected countries, with more than 110,000 cases on April 1, 2020 [3]. Worldwide health systems were reorganized with the aim to both cope with the new disease and maintain essential health service delivery. In this scenario, a concrete risk of health system collapse should be taken into consideration. The large number of patients suffering from respiratory distress syndrome led to an inevitable modification of daily clinical and surgical activity. Various international surgical societies constantly update their recommendations in order to adapt surgical activity on current conditions [4–8]. Italian investigators have already published their experience and the consequent suggestions for general surgery [9, 10]. Unanimously, the goal is to provide timely surgical care to patients presenting with urgent and emergent conditions while optimizing patient care resources and preserving the health of caregivers. However, the current impact of the COVID-19 outbreak on emergency surgery practice is still not investigated. The Associazione Chirurghi Ospedalieri Italiani (Italian Association of Hospital Surgeons, ACOI) is the larger surgical society in Italy, collecting more than 2800 general surgeons from all over the country. It represents, therefore, an ideal background for a survey aimed to show a snapshot of the current practice of emergency surgery in Italy, and our country represents a model of great interest for many countries with similar social-health organization.

## Methods

A 37-item questionnaire was sent to 150 Italian chiefs of General Surgery Units within the ACOI network. A small number of academic surgical units with interest in emergency surgery were also invited. The questionnaire was structured in 4 sections: [1] hospital trait, size, and mission; surgical unit, operating rooms, and intensive care unit (ICU) hallmark; and resources dedicated to COVID-19 patients [2]. Surgical team changes in human resources, duty, and work plan; surgeon infection and quarantine; and responsibility for strategic decisions [3]. Emergency interventions in COVID-19 patients, as per number, indications, surgical presentation, surgical approach, and post-operative complications [4]. Supply of personal protective equipment (PPE), asymptomatic surgeons and/or surgical patient investigation for COVID-19 infection, strategy for limiting the spread of epidemic into the hospital, and intraoperative measures for operating room workers protection. A 48-h time was given for filling the questionnaire. All quantitative values were expressed as mean ± standard deviation (SD), 95% confidence intervals (95% CI), and median and categorical data with percentage frequencies. Differences in continuous data were analyzed using non-parametric tests (Mann-Whitney *U* or Kruskal-Wallis tests as indicated).

## Results

Eighty-two questionnaires were returned and reviewed. Nine centers were excluded because of not performing emergency surgery, and 2 questionnaires resulted incomplete. Therefore, answers from 71 Italian general surgery units practicing emergency surgery [11] were analyzed (compliance 47.3%): 35 from the northern regions and 36 from the central-southern regions. All but 2 questionnaires came from public hospitals (Table 1). In 80.3% of cases, the hospital was accepting COVID-19-positive patients, and 9.8% of them were exclusively dedicated to COVID-19 cases.

**Table 1:** Surgeons and institutions participating in the survey

		Number	Percent
Type of hospital	Public, academic	7	9.8
	Public, non-academic	62	87.3
	Private	2	2.8
Region	Northern Italy	35	49.3
	South-Central Italy	36	50.7
Total bed number	< 201	21	29.6
	201–500	28	39.4
	501–1000	17	23.9
	> 1000	5	7.1
ICU beds	< 10	39	54.9
	10–30	24	33.8
	31–50	6	8.4
	> 50	2	2.8
COVID cases	No	17	23.9
	Yes, COVID and no-COVID cases	47	66.2
	Yes, only COVID cases	7	9.8
Estimated COVID cases on March 29, 2020	< 10	15	21.1
	11–30	10	14.1
	31–60	15	21.1
	> 60	31	43.7
Estimated ICU COVID cases on March 29, 2020	< 10	31	43.7
	11–30	33	46.5
	31–50	3	4.2
	> 50	4	5.6

The overall number of staff surgeons remained unchanged in 76.1% of hospitals, but in 46.5% of surgical departments, one or more surgeons were moved to the Internal Medicine or Emergency Department (ED). Shifts in the work plan were planned in 69.1% of centers in order to minimize the risk of infection. At least one surgeon resulted to be tested positive for COVID-19 in 28.2% of the centers, and in 33.8%, at least one team member was quarantined (Table 2).

**Table 2:** Influence of the COVID-19 epidemic on the surgical team

		Number	Percent
Strategy facing the emergency	Shared with hospital management	34	47.8
	Independently settled by dept. chair	6	8.4
	Imposed by the hospital management	31	43.6
Number of surgeons	Increased	1	1.4
	Reduced	16	22.5
	Unchanged	54	76.1
Surgeons allocated to other departments	Yes	33	46.5
	No	38	53.5
Change in the work plan (forced vacations)	Yes	49	69.0
	No	22	30.9
Team surgeon infected by SARS-Cov2	Yes	20	28.2
	No	51	71.9
Team surgeon on quarantine	Yes	24	33.8
	No	47	66.2

In 23 out of 57 hospitals admitting COVID-19 patients, surgery was carried out in COVID-19 patients (40.3%). The vast majority of surgeons operated only on urgent cases (73.9%), while 21.7% continued to perform a limited number of elective interventions for oncological diseases. The laparoscopic approach was used in 69.6% of cases on COVID-19 patients. The number of urgent interventions significantly dropped after the introduction of mobility restricting measures by the government (Table 3). Approximately 40% of surgeons reported an unusual delay in the presentation of non-traumatic abdominal emergencies. In all these cases, fever had been present for several days before hospital admission. Delay was partially related to patient choice, preferring to stay at home until worsening of the symptoms, and partially due to the waiting list for the COVID-19 test at the emergency room. Only 7% of surgeons reported the occurrence of unexpected complications in COVID-19 patients, with special reference to septic and respiratory complications (Table 4).

**Table 3:** Number of urgent surgical procedures performed in participating surgical centers

Period	Overall number.	Mean ± SD	Median	95%IC	Range	p*
March 2019	2090	29.8 ± 22.3	23	24.5–35.2	1–94	< 0.0001
2020(1)	2190	31.7 ± 15.7	24	22.3–41.2	1–102	
2020(2)	1086	15.7 ± 14.9	10	12.1–19.3	0–80	

*SD* standard deviation; *95%IC* 95% interval confidence; *2020* [1] January 23 to February 22, 2020; *2020* [2] February 23 to March 22, 2020

*March 2019–2020 [1], *p* = 0.826; 2020 [1]–2020 [2], *p* < 0.0001

**Table 4:** Emergency surgery in COVID-19 + cases

		Number	Percent
Surgical interventions in COVID-19+	No	48	67.6
	Yes	23	32.4
Indications to surgery^1^	Very urgent diseases^2^	16	22.5
	Urgent diseases^3^	11	15.5
	Trauma	3	4.2
	Elective oncologic surgery	4	5.6
Surgical approach	Only laparoscopy	5	7.0
	Only open	8	11.3
	Open and laparoscopy	10	14.1
Comparison with the number of February–March 2019 emergency interventions	Increased	8	11.3
	Reduced	39	54.9
	Stable	24	33.8
Unusual delay in presentation for urgent pathology^4^	Yes	28	39.4
	No	43	60.6
Increase in post-operative complication rate	Yes	5	7.1
	No	66	92.9

^1^In each center, more than 1 answer was reported

^2^For example, bowel perforations, diffuse peritonitis, and septic shock, hemorrhages with shock, bowel ischemia, and necrosis

^3^For example, acute appendicitis with localized peritonitis, acute cholecystitis, and obstruction

^4^For example, unusual high rate of acute gangrenous appendicitis, perforated cholecystitis, and stanched bowel perforation

While in 100% of cases, epidemic spread containing measures was put in place for hospital visitors and patients, and adequate strategies to protect health care workers against infection were suboptimal with respect to the WHO recommendations in 70.4% of hospitals [5]. In addition, nasopharyngeal swabs (NS) were performed on symptomatic surgeons only and were not performed at all in 77.5% and 12.7%, respectively, of hospitals. Similarly, NS were performed only on symptomatic patients and none of the surgical patients in 67.6% and 16.9%, respectively, of hospitals. Finally, advanced personal protective equipment (PPE) for operating room workers was adopted for all interventions in only 12.7% of hospitals and measures to reduce the dispersion of biological aerosol during minimally invasive operations were adopted in only 35.2% of the operating rooms (Table 5).

**Table 5:** PPE supply and utilization

		Number	Percent
Institutional measures for visitors	Yes	71	100
	No	0	0
Institutional measures for patients	Yes	71	100
	No	0	0
Institutional measures for surgeons	Yes	50	70.4
	No	21	29.6
Swabs on operated patients	Yes, all operated patients	11	15.5
	Nobody	12	16.9
	Only for symptomatic	48	67.6
Swabs on surgeons	Yes, all surgeons	7	9.8
	Nobody	9	12.7
	Only for symptomatic	55	77.5
Advanced PPE for professionals in OR during surgery	Yes, all cases	9	12.7
	Never	22	30.9
	Only for documented COVID+ pts	40	56.3
Measures to reduce the dispersion of biological aerosol during MIS	Yes	25	35.2
	No	46	64.8
	Only for documented COVID+ pts	0	0

*MIS* minimally invasive surgery

## Discussion

While international scientific societies are rapidly producing recommendations dealing with COVID-19 outbreak [4–8], a starting point is useful in order to frame the operating areas that deviate most from the recommendations, on which it will be necessary to insist more. We performed the current inquiry during the peak of the COVID-19 outbreak in Italy (March 26–28, 2020), giving a very short answering time (48 h) in order to gather a true “snapshot” of the situation. Although the overall compliance rate (43.7%) was low, it nonetheless represents a good sample of Italian general surgery units.

First, the COVID-19 pandemic has an enormous impact on surgery: over 80% of surgical departments changed their practices, 70% shifted work plans, and in 1 out of 2 of them, some team members were allocated to non-surgical departments. About 1 out of 3 surgical departments had team members who were sick or in preventive quarantine. This confirms that the pandemic extensively affects the surgical activities, as already stated [12]. It is therefore strongly advisable that international scientific societies develop unequivocal recommendations which do not contrast with each other.

Focusing on urgent interventions, about half of the centers reported a drop in the overall number of urgent cases (Table 3), however, associated with a more severe presentation due to a diagnostic delay. Many patients with fever are asked by the authorities not to go to the hospital if they do not have breathing difficulties. In these patients, the fever may not be caused by COVID-19-related pneumonia but by an abdominal infection. There are also a number of patients whose diagnostic delay is linked to the time spent in the emergency room, both because of the scarcity of available hospital beds and because of the diagnostic work-up for COVID-19. Thus, it may be useful that recommendation statements devote attention also to the pre-hospital phase and to the management of patients in the emergency room.

The surgical attitude does not seem to have changed significantly in COVID-19 patients, with particular reference to the use of the laparoscopic approach. Indeed, over 2 out of 3 patients were operated on with a minimally invasive technique. The submitted questionnaire does not allow a detailed analysis of post-operative results. However, only 7% of surgeons reported unexpected post-operative complications. Pulmonary complications and unexpected fever were the most common complications reported in positive-tested COVID-19 patients who undergone surgery irrespectively to the type of surgery and surgical approach. This is in partial contrast to recent observations, nevertheless reporting on a very small number of cases [13]. It seems important that recommendations generated by the international scientific societies take into account the observation of Italian general surgeons that the laparoscopic approach does not significantly worsen the outcomes. Recommendations against the use of laparoscopy seem to be linked more to theoretical and physio-pathological considerations than to real data. It is likely that these recommendations, conflicting with the personal opinion of most surgeons, will be little applied in daily practice, generating a significant amount of malpractice claims.

With regard to PPE supply and utilization, there is a significant gap between official recommendations [14–17] and daily clinical practice. In more than 1 out of 4 hospitals, no measures to reduce the health workers’ infections are reported. In almost all centers, NS are performed only on symptomatic surgeons and only on symptomatic surgical patients. In less than 1 out of 10 and 1 out of 6 cases, NS are performed on all surgeons and all surgical patients, respectively. Finally, intraoperative recommendations [18, 19] are very rarely respected in our survey. Recommendations on this issue must be based on incontrovertible data and not on theoretical considerations; otherwise, a large number of legal complaints will be promoted by health professionals against the hospitals.

## Conclusions

Two main considerations arise from the data of this survey. Contraction of admissions for urgent and emergent conditions in the first period of lockdown could be followed by a rebound surge of a large amount of patients with complicated acute diseases quickly overloading responsiveness of the few human resources and the already crowded intensive care units and surgical wards. In Italy, a few number of centers are in condition to adopt the scientific society guidelines to support patient and health care workers’ safety. Both situations require new strategies to improve the health system response to the emergency into an emergency that is reasonably here to come [20]. The at-home patient care model should be implemented in order to identify patients requiring emergency surgery. General practitioners should be appointed to select patients before referral to ED and be furnished of all the PPE that are still scarce for general practice. Physical examination and basic diagnostic procedures (abdominal, chest ultrasound, and NS) settled at home are of paramount importance for patient selection before access to the ED where, to date, time for diagnostic and therapeutic procedures is prolonged waiting for the results of NS. Efforts to maintain in the ED separate COVID-free pathways that guarantee basic assistance for urgent diseases are another element to speed up the treatment of emergency cases and mitigate the patients’ fear of being infected in the hospital.

## Data Availability

Not applicable.

## Abbreviations

ED: Emergency department
ICU: Intensive care unit
MIS: Minimally invasive surgery
NS: Nasopharyngeal swabs
PPE: Personal protective equipment
WHO: World Health Organization
